# Mental health and substance use among women and men at the intersections of identities and experiences of discrimination: insights from the intersectionality framework

**DOI:** 10.1186/s12889-019-6430-0

**Published:** 2019-01-23

**Authors:** Milkie Vu, Jingjing Li, Regine Haardörfer, Michael Windle, Carla J. Berg

**Affiliations:** 1 Department of Behavioral Sciences and Health Education, Rollins School of Public Health, 1518 Clifton Rd NE, Atlanta, GA 30322 USA; 2 Winship Cancer Institute, 1365 Clifton Rd, Atlanta, CA 30322 USA

**Keywords:** Intersectionality, Racial discrimination, African-Americans, Sexual orientation discrimination, Substance use, Mental health, Young adult college students, Word count (manuscript body): 4924 words.

## Abstract

**Background:**

Intersectionality theory focuses on how one’s human experiences are constituted by mutually reinforcing interactions between different aspects of one’s identities, such as race, class, gender, and sexual orientation. In this study, we asked: 1) Do associations between intersecting identities (race and sexual orientation) and mental health (depressive symptoms) and substance use (alcohol, tobacco, and marijuana) differ between men and women? and 2) How do single or intersecting self-reports of perceived racial and/or sexual orientation discrimination influence mental health and substance use outcomes for men and women? We compared results of assessing identities versus experiences of discrimination.

**Methods:**

Multivariable regressions were conducted on cross-sectional data from 2315 Black and White college students. Predictors included measures of sociodemographic characteristics and experiences of discrimination. Outcomes included past 2-week depressive symptoms (PHQ-9), past 30-day alcohol use, past 30-day tobacco use, and past 30-day marijuana use.

**Results:**

Intersecting identities and experience of discrimination had different associations with outcomes. Among women, self-reporting both forms of discrimination was associated with higher depressive symptoms and substance use. For example, compared to women experiencing no discrimination, women experiencing both forms of discrimination had higher depressive symptoms (B = 3.63, CI = [2.22–5.03]), alcohol use (B = 1.65, CI = [0.56–2.73]), tobacco use (OR = 3.45, CI = [1.97–6.05]), and marijuana use (OR = 3.38, CI = [1.80–6.31]). However, compared to White heterosexual women, White sexual minority women had higher risks for all outcomes (B = 3.16 and CI = [2.03–4.29] for depressive symptoms, B = 1.45 and CI = [0.58–2.32] for alcohol use, OR = 2.21 and CI = [1.32–3.70] for tobacco use, and OR = 3.01 and CI = [1.77–5.12] for marijuana use); while Black sexual minority women had higher tobacco (OR = 2.64, CI = [1.39–5.02]) and marijuana use (OR = 2.81, CI = [1.33–5.92]) only. Compared to White heterosexual men, White sexual minority men had higher depressive symptoms (B = 1.90, CI = [0.52–3.28]) and marijuana use (OR = 2.37, CI = [1.24–4.49]).

**Conclusions:**

Our results highlight the deleterious impacts of racial discrimination and sexual orientation discrimination on health, in particular for women. Future studies should distinguish between and jointly assess intersecting social positions (e.g., identities) and processes (e.g., interpersonal experience of discrimination or forms of structural oppression).

## Background

### Application of intersectionality theory in public health research

Intersectionality theory highlights how one’s human experiences are constituted by mutually reinforcing interactions between different aspects of one’s identities, such as race, class, gender, and sexuality [1]. Intersectionality as an analytical concept for the social and health sciences has its roots in black feminist scholarship. In the seminal article “Mapping the margins: Intersectionality, identity politics, and violence against women of color,” Crenshaw pointed out that “the intersection of racism and sexism factors into Black women’s lives in ways that cannot be captured wholly by looking at the race or gender dimensions of those experiences separately” [2]. While Crenshaw focused mainly on the intersection of sex and race, she also highlighted the need to investigate aspects of one’s identity that extend beyond these two categories, as well as the ways in which these intersecting aspects create lived experiences embedded in structural systems of opportunities and oppression.

Crenshaw’s arguments are further extended in works by others such as Bowleg (2008; 2012) and Bauer (2014). For example, Bowleg asserted that intersectionality should be applied as an analytical theoretical framework as opposed to a traditional theory with operationalized and empirically testable variables [3, 4]. Additionally, the core tenet and starting point of intersectionality should be “multiple socially disadvantaged statuses,” or in other words, historically oppressed and marginalized populations such as racial or sexual minority populations [4]. In regard to modeling intersectionality through statistical methods, Bauer emphasized the need to distinguish between variables capturing intersecting identities (e.g., race or sexual orientation) and positions (e.g., racism or homophobia), as well as the need to structure statistical models properly to make differential effects visible across strata or groups [5]. While intersectionality arises from the more historical, interpretative, and qualitative perspectives in the social sciences [6], the abovementioned works have been fundamental in providing more concrete theoretical and methodological directions for quantitatively modeling intersectionality in public health research.

Recent years have seen a proliferation in the number of health studies using intersectionality as a guiding framework along with increasing appreciation of its potential for investigating social determinants of health [7]. Intersectionality has been explicitly applied in research examining disparities or differences in a variety of health beliefs, behaviors, and outcomes, including mental health [6, 8] and substance use [9]. In addition to examining the intersection of identity categories typically seen in public health, such as age, sex, race, sexual orientation, and socioeconomic status, recent studies have also incorporated other measures accounting for social phenomena that reflect power and oppression [10]. Intersections of different experiences of discrimination have also received increasing attention [11, 12].

### The relationship between discrimination and mental health and substance use

Central to the intersectionality theory is the idea that multiple social identities at the micro-level (e.g., race, sex, or social class) are linked to macro- and structural-level inequalities (e.g., racism, sexism, and poverty) [3, 4]. This idea is shared with the *minority stress theory*. Minority stress theory posits that individuals with membership in a minority group will experience unfair treatment due to their group membership [13–15]. Similar to intersectionality theory, minority stress theory hypothesizes that discrimination plays an important role in explaining health disparities between dominant and minority groups.

Several pathways have been proposed for how interpersonal discrimination negatively affects health outcomes and increases health risk behaviors. In regard to mental health, interpersonal and direct experiences with discrimination can lead to heightened vigilance, challenge one’s beliefs about fairness and justice, create internalized stigma towards oneself, and exacerbate physiological and psychological stress, all of which contribute to poorer mental health outcomes [14, 16–18]. A meta-analysis of 110 studies found a significant negative correlation between experiences of perceived discrimination and mental health status [19].

Regarding substance use (e.g., alcohol, tobacco, and marijuana), experiences of discrimination can prompt adolescents and young adults to increase their affiliation with drug-using peers, which subsequently may lead to higher substance use [20]. In addition, individuals exposed to discrimination are more likely to endorse substance use as a coping mechanism [21, 22]. Moreover, psychological distress as a result of experiences of discrimination has also been shown to play a mediating role in the relationship between discrimination and substance use [23–25].

### Issues in the application of intersectionality

Two interrelated challenges appear in the application of intersectionality in public health. The first issue is the tension between the *intersectionality paradox* and the *multiple jeopardy* perspective (also known as *multiple disadvantages* or *multiple-hierarchy stratification* approach). Bowleg emphasized the “intersectionality paradox” as one of the most noteworthy contributions of intersectionality theory to public health [4]. According to Bowleg, “low”-status social identities (e.g., racial or sexual minority) do not automatically equate disadvantages; rather, they intersect with “high”-status identities (e.g., high socioeconomic status) to produce differences in outcomes. An example of the “intersectionality paradox” could be seen in Rosenfield’s study on mental health at the intersection of sex, race, and class [8]. The study demonstrated that, among women with higher than a high school education, Black women have lower rates of depression than White; among women with lower education levels, there were no significant differences between the two groups. These different patterns for women were not indicated in Black versus White men in the study.

While the multiple jeopardy approach also investigates the impacts of multiple social identities and statuses, this perspective assumes that disadvantages accumulate in an added-burden or additive fashion. With additional minority statuses, individuals are assumed to be at risk for incrementally poorer health [14, 26–28]. For example, at the intersections of race, sex, and sexual orientation, a woman who is Black and sexual minority is assumed to have worst health outcomes (i.e., three “low”-status identities), whereas a man who is White and heterosexual would have best health outcomes (i.e., three “high”-status identities). In recent years, scholars have identified discrepancies in findings for the multiple jeopardy and criticized this approach for oversimplifying social realities [8, 29], though there still remains a need for more empirical data to address these issues.

The tension between the intersectionality and multiple jeopardy approaches also points to a need to reevaluate measurements in public health and, in particular, the need to distinguish between identity and experience. Frequently, studies in this research area do not include measures for discrimination, and many studies still treat disadvantaged statuses or minority identities as identical to, or an approximation for, experiences of discrimination. For example, a commonly seen approach is when studies report a poorer health outcome or increased health risks for a minority group, they often hypothesize that the differences may be due to impacts of interpersonal discrimination, without actually including a measure for discrimination [30, 31]. While it is logical and consistent with minority stress theory to posit that individuals with minority statuses will face stigma due to their membership in the minority groups, it is problematic to assume that these two domains (identity and experience) are interchangeable or synonymous.

In light of the tensions of how intersectionality has been developed and applied in public health, this paper seeks to contribute to the field by investigating the following two questions: 1) Do associations between intersecting identities (i.e. race and sexual orientation) and mental health (depressive symptoms) and substance use (alcohol, tobacco, and marijuana) differ between men and women? and 2) How do single or intersecting self-reports of perceived racial and/or sexual orientation discrimination influence mental health and substance use outcomes for men and women? In answering these two questions, we compare results of assessing identities versus experiences of discrimination on health behaviors and outcomes. The goal of this research is to provide empirical evidence supporting the need to: 1) incorporate understanding of differing mental health and substance use outcomes based on intersections of identities and intersections of experiences of discrimination, and 2) distinguish between the two domains of identity and experience in public health research.

## Methods

### Study design and participants

Data for the current study came from Project DECOY (**D**ocumenting **E**xperiences with **C**igarettes and **O**ther Tobacco in **Y**oung Adults). The methods employed for sampling and recruitment for Project DECOY have been described elsewhere [32]. Briefly, this is a two-year, six-wave longitudinal cohort study that involved 3418 racially/ethnically diverse students (ages 18 to 25 years) from seven colleges and universities in Georgia. Schools are located in both rural and urban settings and include two public universities/colleges, two private universities, two community/technical colleges, and one historically black university. Our project was approved by the Emory University and ICF Institutional Review Boards (IRBs) as well as the IRBs of the participating colleges and universities. Data collection began in Fall 2014 and consisted of self-report assessments via an online survey every four months for two years (during Fall, Spring, and Summer).

The registrar’s office from each campus provided e-mail addresses for English-speaking students ages 18–25. We randomly selected 3000 email addresses from each of the three largest campuses, and emailed a census of students at the four smaller campuses with fewer than 3000 students. We met our sampling quota target in a short time interval (24 h at the private schools to seven days at the technical colleges) and enrolled between 12.0 to 59.4% of those approached at different campuses, and overall 22.9% (*N* = 3574/15,607). Seven days after initial recruitment and completion of the baseline survey, we asked participants to confirm their participation by clicking a “confirm” button included in an email sent to them. The email reiterated the tasks involved in the study and its timeline. Once participants clicked “confirm,” they were enrolled into the study and sent their first incentive in the form of a $30 gift card via email. The confirmation rate was 95.6% (*N* = 3418/3574). Our intent was to enroll participants who were engaged in email and were potentially more likely to be retained in the subsequent waves of the larger, multi-wave longitudinal project.

The current analyses examined data from Wave 5 of the study. Data were collected between April and May 2016, which took place around 1.5 years from the baseline (Wave 1) data collection. Wave 5 surveys were completed by 2690 participants (the retention rate from Wave 1 to Wave 5 is 78.7%). Since our research questions focused on participants who identified as Black or White, a total of 2315 participants who met this criterion were included in the analyses.

### Measures

Data were taken from the baseline survey assessments of socio-demographic information and the Wave 5 assessment of experience of discrimination, depressive symptoms, and use of alcohol, tobacco, and marijuana within the past 30 days.

#### Sociodemographic Characteristics

Sociodemographic factors captured in the surveys included sex (0 = Male, 1 = Female), race (0 = White, 1 = Black), sexual orientation (0 = Heterosexual, 1 = Sexual minority), age, highest level of education attained by parents (0 = Bachelor’s degree & above, 1 = Below a Bachelor’s degree), and school type (0 = Private school, 1 = State university, 2 = Technical college, and 3 = Historically black college/university (HBCU)).

#### Experience of discrimination

Experience of discrimination were assessed with: “How often have you felt as though you were treated badly because of your race or ethnicity?” and “How often have you felt as though you were treated badly because of your sexual orientation?” Response options for both questions ranged from 1 = Never to 5 = Very often. These questions were adapted from measures used in previous works on racial and ethnic discrimination in youth in the United States [33, 34].

Sensitivity analyses were conducted to see if results differed significantly when the variables were operationalized as ordinal variables (score ranging from 1 to 5) and as binary categorical variables (ever versus never having any experience with discrimination). Because results did not significantly change, we conceptualized the discrimination variables as binary categorical variables and dichotomized responses to either 0 = never having any experience with discrimination or 1 = ever having any experience with discrimination.

#### Outcome: Depressive symptoms

Depressive symptoms were assessed using the Patient Health Questionnaire-9 (PHQ-9), a nine-item assessment scale using diagnostic criteria for depressive disorders from the Diagnostic and Statistical Manual of Mental Disorders (DSM)-IV [35]. The items asked participants whether they had been bothered by any of the listed problems (e.g., “feeling down, depressed, or hopeless”) during the previous two weeks. Response options ranged from 1 = Not at all to 4 = Nearly every day. Summed scores across all nine items were created for each participant. Cronbach’s alpha for the PHQ-9 was 0.90.

In our sample, any participant who chose “Refuse” on an item in the PHQ-9 scale was coded as having missing data for that item. For the PHQ-9, it had been suggested that if participants had missing data for one or two out of the nine items, the missing values should be substituted with the average score of the non-missing items. Participants with missing data for more than two items should be coded as having missing data for the summed scores [36]. All participants with missing PHQ-9 data in our sample (*n* = 31) had missing data for more than two items and were all coded as having missing data for the summed score.

#### Outcome: Use of alcohol

Use of alcohol was assessed with: “In the past 30 days, on how many of those days did you drink alcohol?” Response options ranged from 0 to 30 days.

#### Outcome: Use of tobacco products

Use of tobacco products was assessed with: “During the past 30 days, on how many days did you: smoke cigarettes; smoke little cigars or cigarillos; use a smokeless tobacco product; use an e-cigarette; or use a hookah or waterpipe?” Given the highly right-skewed distribution of this variable, responses were dichotomized into 0 = no days of use for all products or 1 = one or more days of use of any tobacco product.

#### Outcome: Use of marijuana

Use of marijuana was assessed with: “During the past 30 days, on how many days did you use marijuana?” Given the highly right-skewed distribution of this variable, responses were dichotomized into 0 = no day of use or 1 = one or more days of use.

### Data analysis

Simple logistic regressions were conducted to assess the association between race and experience of racial discrimination as well as the association between sexual orientation and experience of sexual orientation discrimination. We found that being Black was associated with reporting racial discrimination (odd ratio or OR = 7.46, *p* < 0.001), and being sexual minority was associated with reporting sexual orientation discrimination (OR = 42.67, *p* < 0.001). We also conducted bivariate analyses to examine sociodemographic characteristics and experience of discrimination in relation to the four outcomes of interest: 1) number of depressive symptoms; 2) number of days of alcohol use within the past 30 days; 3) use of any tobacco products within the past 30 days; and 4) use of marijuana within the past 30 days.

Multivariable linear and binary logistic regression models were used to identify correlates of the outcomes of interest. Given evidence on sex differences in depression and substance use behaviors (refer to Table 1), for each outcome, we stratified models by sex. We then constructed two different multivariable regression models (for a total of four models per outcome). For each outcome, Model 1 contained variables capturing intersecting identities (White and heterosexual, White and sexual minority, Black and heterosexual, or Black and sexual minority), and Model 2 contained variables capturing experience of discrimination (no experience, only racial discrimination, only sexual orientation discrimination, or both forms of discrimination). Key sociodemographic variables (age, highest parental education, and school type) were also entered into all models. In our data, we observed that 70.91% of Black students reported experiencing racial discrimination compared to 24.63% of White students; moreover, 2.85% of heterosexual students reported experiencing sexual orientation discrimination compared to 55.56% of sexual minority students. We also observed a high correlation between race and experience of racial discrimination as well as between sexual orientation and experience of sexual orientation discrimination (OR = 7.46 and OR = 42.67, respectively, with both p values < 0.001, as noted above). Therefore, we did not include race and experience of racial discrimination in the same model. We also did not include sexual orientation and experience of sexual orientation discrimination in the same model. Analyses were conducted using SAS 9.4.

**Table 1:** Characteristics of study participants in relations to sex (bivariate analyses)

Characteristics	Total Sample *N* = 2315 N (%) or M (SD)	Women (N = 1522) N (%) or M (SD)	Men (*N* = 793) N (%) or M (SD)	p
Age (SD)	20.49 (1.91)	20.50 (1.89)	20.48 (1.97)	.84
Sex				
Male	793 (34.25%)			
Female	1522 (65.75%)			
Race				<.001
White	1734 (74.90%)	1047 (68.79%)	687 (86.63%)	
Black	581 (25.10%)	475 (31.21%)	106 (13.37%)	
Sexual orientation^a^ (*N* = 2290)				.45
Heterosexual	2038 (89.00%)	1334 (88.64%)	704 (89.68%)	
Sexual minority	252 (11.00%)	171 (11.36%)	81 (10.32%)	
Highest parental education^b^ (N = 2290)				<.001
Bachelor’s degree or above	1222 (53.36%)	713 (47.31%)	509 (65.01%)	
Below a Bachelor’s degree	1068 (46.64%)	794 (52.69%)	274 (34.99%)	
School type				<.001
Private college/university	940 (40.60%)	564 (37.06%)	376 (47.41%)	
State university	669 (28.90%)	371 (24.38%)	298 (37.58%)	
Technical college	423 (18.27%)	335 (22.01%)	88 (11.10%)	
HBCU	283 (12.22%)	252 (16.56%)	31 (3.91%)	
Experience of racial discrimination	Yes = 839 (36.24%)	Yes = 603 (39.62%)	Yes = 236 (29.76%)	<.001
Blacks reporting discrimination = 412 (70.91% of Blacks)				
Whites reporting discrimination = 427 (24.63% of Whites)				
Experience of sexual orientation discrimination	Yes = 203 (8.77%)^#^	Yes = 131 (8.61%)	Yes = 72 (9.08%)	.70
Sexual minority reporting discrimination = 140 (55.56% of sexual minority students)				
Heterosexual students reporting discrimination = 58 (2.85% of heterosexual students)				
Depression score (*N* = 2284)^d^	5.24 (5.62)	4.65 (5.31)	5.55 (5.75)	<.001
Days of using alcohol within past 30 days	3.58 (4.99)	3.13 (4.51)	4.45 (5.69)	<.001
Use of any tobacco products* within past 30 days				<.001
Yes	399 (17.24%)	217 (14.26%)	182 (22.95%)	
Use of marijuana within past 30 days^c^ (*N* = 2220)				.31
Yes	278 (12.52%)	175 (12.01%)	103 (13.50%)	

* Includes any use of cigarettes, little cigars/cigarillos, smokeless tobacco, e-cigarettes, or hookahs within the past 30 days

^a^ Data are missing for 25 participants who chose “Refuse” ^b^ Data are coded as missing for 25 participants who chose “Don’t know”

^c^ Data are missing for 95 participants who chose “Refuse” ^d^ Data are coded as missing for 31 participants who chose “Refuse” on items of the PHQ-9 scale

^#^ Discrepancy in sum is due to 4 students with missing sexual orientation data who reported discrimination

*HBCU* Historically Black Colleges/Universities, *N* Size of the Sample, *M* Mean, *SD* Standard Deviation, *p* Probability Value

## Results

The average age at baseline was 20.49 years (standard deviation or SD = 1.91), 65.75% (*n* = 1522) was female, 25.10% (*n* = 581) was Black, and 11.00% (*n* = 252) was sexual minority. Among women, 7.24% (*n* = 109) were White sexual minority, 4.12% (*n* = 62) were Black sexual minority, and 26.78% (*n* = 403) were Black heterosexual. Among men, 8.15% (*n* = 64) were White sexual minority, 2.17% (*n* = 17) were Black sexual minority, and 11.21% (*n* = 88) were Black heterosexual.

Racial discrimination was reported by 36.24% (*n* = 839), and experience of sexual orientation discrimination was reported by 8.77% (*n* = 203) of participants. The average score on PHQ-9 was 5.24 (SD = 5.62), and the average number of days of using alcohol within the past 30 days was 3.58 (SD = 4.99). Within the past 30 days, 17.42% (n = 399) of participants reported use of tobacco products, and 12.52% (*n* = 278) reported use of marijuana. Table 1 provides additional characteristics of the study participants.

### Depressive symptoms

In bivariate analyses, higher depressive symptoms was associated with being female, sexual minority, reporting racial discrimination, reporting sexual orientation discrimination, younger age, having parents with highest education being below a Bachelor’s degree, and school type.

Multivariable linear regression models (shown in Table 2) indicated that compared to White heterosexual women, White sexual minority women had higher depressive symptoms (beta or B = 3.16, *p* < 0.001). Compared to women who experienced no discrimination, women who experienced only racial discrimination (B = 1.57, *p* < 0.001), only sexual orientation discrimination (B = 2.59, *p* < 0.001), and both forms of discrimination (B = 3.63, *p* < 0.001) had higher depressive symptoms. For women, older age was associated with lower depressive symptoms.

**Table 2:** Multivariable linear regressions on sociodemographic characteristics, intersecting identities, and intersecting experiences of discrimination and outcome of depressive symptoms (per the PHQ-9)

	Women’s depressive symptoms					Men’s depressive symptoms			
	Model with intersecting identities		Model with intersecting experiences of discrimination			Model with intersecting identities	Model with intersecting experiences of discrimination		
	B and CI		p	B and CI	p	B and CI	p	B and CI	p
Age	−0.24 (− 0.40 – − 0.09)		.002	− 0.27 (− 0.43 – − 0.12)	<.001	0.08 (− 0.11–0.27)	.42	0.08 (− 0.11–0.27)	.39
Highest parental education									
Bachelor’s degree or above	Reference			Reference		Reference		Reference	
Below a Bachelor’s degree	0.80 (0.16–1.44)		.01	0.61 (−0.02–1.23)	.06	0.22 (− 0.64–1.08)	.62	0.23 (− 0.62–1.08)	.59
School type									
Private college/university	Reference			Reference		Reference		Reference	
State university	1.10 (0.33–1.87)		.005	0.67 (−0.08–1.43)	.08	0.83 (−0.02–1.68)	.06	0.82 (− 0.02–1.66)	.06
Technical college	−0.05 (− 0.90–0.81)		.91	−0.32 (− 1.17–0.52)	.45	−0.06 (− 1.42–1.30)	.93	0.07 (− 1.25–1.40)	.91
HBCU	−0.37 (− 1.54–0.80)		.54	− 1.62 (− 2.55–0.68)	<.001	−2.05 (− 4.46–0.37)	.10	−1.91 (− 4.06–0.25)	.08
Intersecting identities									
Being White and heterosexual	Reference					Reference			
Being White and sexual minority	3.16 (2.03–4.29)		<.001			1.90 (0.52–3.28)	.01		
Being Black and heterosexual	−0.38 (−1.27–0.50)		.40			0.74 (−0.62–2.10)	.29		
Being Black and sexual minority	0.18 (−1.41–1.77)		.82			1.91 (−0.80–4.63)	.17		
Experience of discrimination									
No discrimination				Reference				Reference	
Only racial discrimination				1.57 (0.93–2.21)	<.001			0.96 (0.08–1.85)	.03
Only sexual orientation discrimination				2.59 (1.11–4.07)	<.001			3.78 (2.00–5.56)	<.001
Both racial and sexual orientation discrimination				3.63 (2.22–5.03)	<.001			1.44 (−0.39–3.27)	.12
	Adj R^2^ = 0.04			Adj R^2^ = 0.05		Adj R^2^ = 0.01		Adj R^2^ = 0.03	

*HBCU* Historically Black Colleges/Universities, *B* Beta, *CI* Confidence Interval, *p* Probability Value, *R*^*2*^ Coefficient of Determination

Compared to White heterosexual men, White sexual minority men had higher depressive symptoms (B = 1.90, *p* = 0.01). Compared to men who experienced no discrimination, men who experienced only racial discrimination (B = 0.96, *p* = 0.03) and only sexual orientation discrimination (B = 3.78, *p* < 0.001) had higher depressive symptoms.

### Use of alcohol

In bivariate analyses, higher number of days of alcohol use within the past 30 days was associated with being male, White, and sexual minority, as well as older age, having parents with highest education of a bachelor’s degree or above, and school type.

Multivariable linear regression models (shown in Table 3) indicated that, compared to White heterosexual women, White sexual minority women had higher alcohol use (B = 1.45, *p* = 0.001), while Black heterosexual women had lower alcohol use (B = − 1.39, *p* < 0.001). Compared to women experiencing no discrimination, women who experienced both racial and sexual orientation discrimination had higher alcohol use (B = 1.65, *p* = 0.003). For both men and women, older age was associated with higher alcohol use. Compared to attending a private school, attending a technical college was associated with lower alcohol use; for women, attending a state university was associated with lower alcohol use. For both men and women, having parents with highest education of less than a Bachelor’s degree was associated with lower alcohol use.

**Table 3:** Multivariable linear regressions on sociodemographic characteristics, intersecting identities, and intersecting experiences of discrimination and outcome of number of days of alcohol use in the past 30 days

	Women’s number of days of alcohol use				Men’s number of days of alcohol use			
	Model with intersecting identities		Model with intersecting experiences of discrimination		Model with intersecting identities		Model with intersecting experiences of discrimination	
	B and CI	p	B and CI	p	B and CI	p	B and CI	p
Age	0.45 (0.33–0.58)	<.001	0.44 (0.32–0.56)	<.001	0.63 (0.43–0.84)	<.001	0.64 (0.44–0.84)	<.001
Highest parental education								
Bachelor’s degree or above	Reference		Reference		Reference		Reference	
Below a Bachelor’s degree	−0.82 (−1.31 – −0.33)	.001	−0.94 (−1.43 – − 0.45)	<.001	−1.25 (−2.14 – − 0.35)	.006	−1.27 (−2.16 – − 0.38)	.005
School type								
Private college/university	Reference		Reference		Reference		Reference	
State university	−0.65 (−1.25 – − 0.06)	.03	− 0.92 (− 1.50 – − 0.33)	.002	-0.33 (− 1.22–0.55)	.46	−0.35 (− 1.23–0.54)	.44
Technical college	−1.17 (− 1.82 – − 0.51)	.001	−1.39 (− 2.04 – − 0.74)	<.001	−2.15 (−3.58 – − 0.73)	.003	−2.28 (−3.68 – − 0.89)	.001
HBCU	−0.45 (− 1.35–0.44)	.32	−1.75 (− 2.46 – − 1.03)	<.001	−0.87 (− 3.34–1.59)	.49	−1.25 (− 3.42–0.92)	.26
Intersecting identities								
Being White and heterosexual	Reference				Reference			
Being White and sexual minority	1.45 (0.58–2.32)	.001			0.94 (−0.51–2.38)	.20		
Being Black and heterosexual	−1.39 (−2.07 – −0.72)	<.001			−0.64 (−2.07–0.78)	.37		
Being Black and sexual minority	0.32 (−0.89–1.53)	.61			0.54 (−2.29–3.37)	.71		
Experience of discrimination								
No discrimination			Reference				Reference	
Only racial discrimination			−0.003 (−0.50–0.49)	.99			0.10 (−0.83–1.03)	.83
Only sexual orientation discrimination			0.37 (−0.78–1.52)	.52			1.14 (−0.74–3.01)	.23
Both racial and sexual orientation discrimination			1.65 (0.56–2.73)	.003			0.50 (−1.40–2.39)	.61
	Adj R^2^ = 0.08		Adj R^2^ = 0.06		Adj R^2^ = 0.07		Adj R^2^ = 0.06	

*HBCU* Historically Black Colleges/Universities, *B* Beta, *CI* Confidence Interval, *p* Probability Value, *R*^*2*^ Coefficient of Determination

### Use of tobacco products

In bivariate analyses, use of tobacco products within the past 30 days was associated with being male, sexual minority, reporting racial discrimination, reporting sexual orientation discrimination, and school type.

Multivariable logistic regression models (shown in Table 4) indicated that, compared to White heterosexual women, White sexual minority women (OR = 2.21, *p* = 0.003) and Black sexual minority women (OR = 2.64, *p* = 0.003) were more likely to use tobacco products. Compared to women who experienced no discrimination, women who experienced only racial discrimination (OR = 1.42, *p* = 0.04) and both racial and sexual orientation discrimination (OR = 3.45, *p* < 0.001) were all more likely to use tobacco products. Additionally, for both men and women, compared to attending a private school, students attending a state university or a technical college were more likely to use tobacco products. Women attending a HBCU also were more likely to use tobacco products.

**Table 4:** Multivariable logistic regressions on sociodemographic characteristics, intersecting identities, and intersecting experiences of discrimination and outcome of use of any tobacco products in the past 30 days

	Women’s use of tobacco products				Men’s use of tobacco products				
	Model with intersecting identities	Model with intersecting experiences of discrimination			Model with intersecting identities	Model with intersecting experiences of discrimination			
	OR and CI	p	OR and CI	p	OR and CI	p	OR	CI	p
Age	1.04 (0.96–1.13)	.32	1.04 (0.97–1.13)	.28	0.98 (0.89–1.07)	.60	0.99	(0.90–1.08)	.76
Highest parental education									
Bachelor’s degree or above	Reference		Reference		Reference		Reference		
Below a Bachelor’s degree	1.06 (0.76–1.49)	.72	1.07 (0.77–1.49)	.68	0.70 (0.47–1.04)	.08	0.71	(0.48–1.04)	.08
School type									
Private college/university	Reference		Reference		Reference		Reference		
State university	1.84 (1.19–2.86)	.006	1.72 (1.12–2.65)	.01	1.57 (1.07–2.30)	.02	1.58	(1.07–2.31)	.02
Technical college	3.00 (1.93–4.66)	<.001	2.79 (1.80–4.32)	<.001	2.31 (1.29–4.14)	.005	2.13	(1.20–3.78)	.01
HBCU	1.98 (1.10–3.57)	.02	1.87 (1.14–3.04)	.01	2.64 (0.93–7.46)	.07	1.95	(0.79–4.80)	.15
Intersecting identities									
Being White and heterosexual	Reference				Reference				
Being White and sexual minority	2.21 (1.32–3.70)	.003			0.96 (0.51–1.79)	.89			
Being Black and heterosexual	1.16 (0.75–1.78)	.51			0.64 (0.34–1.24)	.19			
Being Black and sexual minority	2.64 (1.39–5.02)	.003			1.06 (0.33–3.35)	.93			
Experience of discrimination									
No discrimination			Reference				Reference		
Only racial discrimination			1.42 (1.02–1.98)	.04			0.85	(0.57–1.27)	.43
Only sexual orientation discrimination			1.51 (0.71–3.21)	.28			0.79	(0.34–1.87)	.59
Both racial and sexual orientation discrimination			3.45 (1.97–6.05)	<.001			1.20	(0.55–2.61)	.64

*HBCU* Historically Black Colleges/Universities, *OR* Odd Ratio, *CI* Confidence Interval, *p* Probability Value

### Use of marijuana

In bivariate analyses, use of marijuana within the past 30 days was associated with being Black, sexual minority, reporting racial discrimination, reporting sexual orientation discrimination, and school type.

Multivariable logistic regression models (shown in Table 5) indicated that, compared to White heterosexual women, Black heterosexual women (OR = 1.72, *p* = 0.02), Black sexual minority women (OR = 2.81, *p* = 0.007), and White sexual minority women (OR = 3.01, *p* < 0.001) were all more likely to use marijuana. Compared to women who experienced no discrimination, women who experienced racial (OR = 1.48, *p* = 0.03) or sexual orientation discrimination (OR = 3.07, *p* = 0.001) or both forms of discrimination (OR = 3.38, *p* < 0.001) were more likely to use marijuana. Compared to White heterosexual men, White sexual minority men (OR = 2.37, *p* = 0.009) were more likely to use marijuana. Finally, compared to attending a private school, women attending a state university were more likely to use marijuana. In men, however, this effect was reversed, such that men attending a state university were less likely to use marijuana.

**Table 5:** Multivariable logistics regressions on sociodemographic characteristics, intersecting identities, and intersecting experiences of discrimination and outcome of use of marijuana in the past 30 days

	Women’s use of marijuana				Men’s use of marijuana			
	Model with intersecting identities		Model with intersecting experiences of discrimination		Model with intersecting identities		Model with intersecting experiences of discrimination	
	OR and CI	p	OR and CI	p	OR and CI	p	OR and CI	p
Age	1.00 (0.92–1.10)	.93	1.00 (0.92–1.10)	.96	0.92 (0.82–1.03)	.13	0.91 (0.82–1.02)	.11
Highest parental education								
Bachelor’s degree or above	Reference		Reference		Reference		Reference	
Below a Bachelor’s degree	0.88 (0.62–1.25)	.47	0.88 (0.62–1.25)	.47	1.06 (0.64–1.75)	.82	1.10 (0.67–1.80)	.72
School type								
Private college/university	Reference		Reference		Reference		Reference	
State university	1.91 (1.25–2.94)	.003	1.85 (1.21–2.82)	.004	0.48 (0.29–0.79)	.004	0.48 (0.29–0.79)	.004
Technical college	1.00 (0.59–1.70)	.99	0.99 (0.59–1.66)	.96	0.40 (0.16–1.03)	.06	0.40 (0.16–0.99)	.05
HBCU	1.58 (0.86–2.90)	.14	2.04 (1.24–3.35)	.005	1.29 (0.38–4.36)	.69	1.19 (0.43–3.33)	.74
Intersecting identities								
Being White and heterosexual	Reference				Reference			
Being White and sexual minority	3.01 (1.77–5.12)	<.001			2.37 (1.24–4.49)	.009		
Being Black and heterosexual	1.72 (1.07–2.75)	.02			0.90 (0.37–2.16)	.81		
Being Black and sexual minority	2.81 (1.33–5.92)	.007			3.01 (0.88–10.27)	.08		
Experience of discrimination								
No discrimination			Reference				Reference	
Only racial discrimination			1.48 (1.03–2.13)	.03			1.10 (0.66–1.84)	.71
Only sexual orientation discrimination			3.07 (1.58–5.99)	.001			0.93 (0.31–2.74)	.89
Both racial and sexual orientation discrimination			3.38 (1.80–6.31)	<.001			2.04 (0.86–4.84)	.11

*HBCU* Historically Black Colleges/Universities, *OR* Odd Ratio, *CI* Confidence Interval, *p* Probability Value

## Discussion

This study examines the impacts of intersecting identities versus experiences of discrimination in a sample of young adult college students in Georgia and documents several insightful findings. Results from our study highlight the complex and differential influences of intersecting identities versus intersecting experiences of discrimination on mental health and substance use outcomes. It is clear that in relation to these outcomes, identities and experiences of discrimination do not yield the same effects. Below, in turn, we discuss the implications of our findings for measurement in health disparities research. We also discuss how our findings inform research on experiences of discrimination for women and men. Moreover, we integrate our results with existing studies in the literature looking at the associations between race, sexual orientation, and experiences of discrimination and substance use and mental health.

For example, experiences of both forms of discrimination were associated with worse mental health and higher substance use for women. However, among women, compared to those who identified as White and heterosexual, those who identified as White sexual minority had higher risks for all outcomes, while Black sexual minority had higher odds of using tobacco products and marijuana. Among men, compared to those who identified as White and heterosexual, those who identified as White sexual minority had higher depressive symptoms and odds of using marijuana, but no significant higher risks were observed for Black heterosexual or sexual minority. In addition, we did not find support for the “multiple jeopardy” approach, which asserts that additions of “low”-status identities (e.g., Black or sexual minority) equate incremental disadvantages [27, 28]. Rather, our results are consistent with the “intersectionality paradox” and highlight the complexity when thinking about how “low”-status identities interact with “high”-status identities to produce differences in health behaviors and outcomes [4].

These results have implications for measurement in health disparities research. Researchers need to distinguish between domains of identities and experiences of discrimination by including items capturing intersections within each domain. While we asked about interpersonal experience with discrimination in this study, discrimination can also be conceptualized to include structural inequalities such as lack of access to quality healthcare or internalized racism and homophobia [37, 38].

Our findings contribute to existing literature on the impacts of discrimination on health for college students and young adults. Several studies have documented that perceived racial discrimination is linked to higher depressive symptoms and higher alcohol and tobacco use among Black college students [39–42] and higher alcohol use among White students [43, 44]. Some research has also investigated differential impacts based on sex; for example, a study found that while Black men with more lifetime discrimination had a positive association between daily negative mood and level of nonsocial drinking, this association was reversed in women [45]. In addition, recent research has also examined discrimination encountered by sexual minority students and its link to mental health [46].

Our findings extend this literature by examining effects of different types of discrimination on outcomes among women and men. Our study provides evidence for how experience of discrimination was a strong predictor of higher depressive symptoms and higher substance use for female young adults as well as how patterns of influence of intersecting experience of discrimination on these outcomes differed between male and female young adults. Compared to those experiencing no discrimination, women experiencing a single form of discrimination had higher depressive symptoms and higher odds of using tobacco products and marijuana. Experiencing of both forms of discrimination put women at higher depressive symptoms and higher substance use than experiencing only a single form of discrimination. The effects were not similar among men. Compared to men who did not experience discrimination, those who experienced either racial or sexual orientation discrimination had higher depressive symptoms, but we did not observe any effect of experiencing both forms of discrimination. Thus, research and initiatives to address discrimination and prejudices should pay close attention to the deleterious impacts of discrimination on women’s health, and future studies should continue to investigate effects of different types of discrimination and variations of effects between men and women.

Our results also echo Bauer’s (2014) recommendations for methodological application of intersectionality in public health, in particular through approaches to construct models that make intersectional effects visible to readers [5]. Here we presented one approach to structuring analytical models that helps readers understand how the impact of factors (intersecting identities and experience of discrimination) differed across strata (men and women). Through stratifying our analyses by sex, we found that the differences in all four outcomes along the line of intersecting identities and experience of discrimination were more prominent for women compared to men.

Prior literature has documented more significant risk of substance use for sexual minority women when compared with heterosexual women, and studies also found smaller effect sizes in use among men by sexual orientations [9, 47–50]. For example, data from the National Alcohol Survey suggested that sexual minority adult women had lower alcohol abstention rates and greater odds of reporting alcohol-related problems compared to heterosexual women. The same data showed that few significant differences in use of alcohol and experience of alcohol-related problems existed among men by sexual orientation [49]. Additionally, McCabe and colleagues analyzed data from the National Epidemiologic Survey on Alcohol and Related Conditions [48] to examine substance use and dependence (alcohol, marijuana, and other drugs) and found that the effects of sexual minority status on substance use and substance dependence were larger for women than for men. Moreover, a survey of undergraduate students on drug use showed that sexual minority women were more likely to use marijuana and smoke cigarettes compared to heterosexual women [51]. Among men, the survey found that sexual minority men were less likely to drink more heavily compared to heterosexual men.

Our results are consistent with some of these conclusions in the literature, though, of note, our findings provide novel information because we included racial status in addition to sexual orientation. In our study, while White sexual minority men had higher odds of using marijuana compared to White heterosexual men; no other significant differences were observed for other outcomes or for Black heterosexual and Black sexual minority men. Among women, as mentioned, we found more variations, and also found evidence of sexual minority women with higher risks on a few outcomes compared to heterosexual women (e.g., White sexual minority women had higher use of all substance compared to White heterosexual women, and Black sexual minority women had higher use of tobacco products and marijuana compared to White heterosexual women). However, it should be noted that while some studies found higher or comparable rates of substance use among sexual minority women of color compared to White sexual minority women [9, 52], our study did not replicate this pattern. More research is needed to understand intersectional differences in substance use for women.

### Strengths and limitations

The generalizability of findings is limited because our sample was drawn from young adult college students in Georgia. However, it is important to note that our sample was drawn from diverse schools, including private, public, technical, and historically black colleges and universities in both rural and urban settings. We also limited our analysis to only Black and White students and did not consider non-White Hispanic and Asian students due to the small size of these groups in our sample. Future studies with larger sample size of these populations should continue to investigate the interplay between intersecting identities and experiences of discrimination.

Additionally, in our study, we grouped students identifying as homosexual and students identifying as bisexual into one (“sexual minority”) due to the small sample size of each category. Some research has shown differences in substance use between homosexual versus bisexual college students [53, 54]. Future studies may want to over sample these different categories in order to further understand whether differences exist in regards to mental health and substance use between different sexual minority categories.

While our data was limited by being self-reported, cross-sectional data, the data included key measures, including the assessment of use of different substance (alcohol, diverse alternative tobacco products, and marijuana). Most importantly, as highlighted throughout this paper, the inclusion of items capturing intersecting identities and experience of discrimination allowed us to separate influences of minority statuses versus discrimination on health behaviors and outcomes.

## Conclusions

This study continues the efforts to conceptualize and operationalize intersectionality in public health research and contributes to a developing body of literature that applies intersectionality theory to understand health disparities [3–8]. We provide empirical data to support the “intersectionality paradox” argument and suggest that researchers should not assume that health risks increase with each additional minority status. Future studies should distinguish between and jointly assess intersecting social positions (e.g., identities) and processes (e.g., interpersonal experience of discrimination or forms of structural oppression). Given the large number of potential identities (e.g., sex, race, ethnicity, sexual orientation, class, age, weight, religion, immigration status) and experiences that can be related to these identities, we recommend that researchers use robust theory and evidence to guide their selection of variables. Attention should also be paid to model constructions in order to make intersectional effects visible to readers. Furthermore, subsequent steps to advance intersectionality theory can include investigations of the dynamic nature of identities and how identities or experience of different processes change or remain in different contexts and across lifespan development. We believe such considerations will contribute to the growing field of intersectional health research and further efforts to achieve health equity.

## Abbreviations

B: Beta
DECOY: Documenting experiences with cigarettes and other tobacco in young adults
DSM: Diagnostic and Statistical Manual of Mental Disorders
HBCU: Historically Black Colleges/Universities
IRB: Institutional Review Board
OR: Odd ratio
P: Probability value
PHQ-9: Patient heath questionnaire – 9
SD: Standard deviation
